# Absence of the *mec*A Gene in Methicillin Resistant *Staphylococcus aureus* Isolated from Different Clinical Specimens in Shendi City, Sudan

**DOI:** 10.1155/2015/895860

**Published:** 2015-07-28

**Authors:** Mogahid M. Elhassan, Hani A. Ozbak, Hassan A. Hemeg, Miskelyemen A. Elmekki, Leila M. Ahmed

**Affiliations:** ^1^Department of Med. Laboratory Tech., College of Applied Medical Science, Taibah University, Al-Madinah Al-Munawarah, Saudi Arabia; ^2^Department of Microbiology, College of Medical Laboratory Science, Sudan University of Science and Technology, Khartoum, Sudan; ^3^Department of Microbiology, College of Medical Laboratory Science, Shendi University, Khartoum, Sudan

## Abstract

Absolute dependence on* mec*A gene as the defining standard in determining the resistance of* S. aureus* to methicillin became the subject of distrust by many researchers. The present study aimed to determine the frequency of* mec*A gene in methicillin resistant* S. aureus* (MRSA) isolates using polymerase chain reaction and to correlate its presence to conventional method. In this regard, two hundred* S. aureus* isolates were collected from patients with different diseases attending different hospitals in Shandi City, Sudan. Phenotypic Kirby-Bauer method confirmed the existence of methicillin resistant* S. aureus* in 61.5% of the subjected isolates with MICs ranging from 4 *μ*g/mL to 256 *μ*g/mL when using* E*-test. However, when amplifying a 310 bp fragment of the* mec*A gene by PCR, twelve out of the 123 MRSA isolates (9.8%) were* mec*A negative, whereas all the 77 methicillin sensitive* S. aureus* (MSSA) were* mec*A negative. In conclusion, this study drew attention to the credibility of the* mec*A gene and its usefulness in the detection of all MRSA strains without referring to the traditional methods. Hence, it is highly recommended to consider alternative mechanisms for *β*-lactam resistance that may compete with* mec*A gene in the emergence of MRSA phenomenon in the community.

## 1. Introduction

Methicillin resistant* S. aureus* (MRSA) has become a major public health problem all over the world. It is correlated with incremented morbidity and mortality, compared to other pathogenic bacteria. The elevated colonization rates lead to the incrimination of infection rates in the community and medical centers which leads to a significant increase in treatment cost [1].

The majority of researches in this field suggested that* mec*A gene that is present in all MRSA strains and is known to encode penicillin binding protein 2a (PBP2a), which has a low tropism to all *β*-lactam antibiotics, is the corner stone responsible for producing MRSA phenomenon [2, 3]. Beta-lactam resistance is attributed mostly to mutations in the* mec*A gene, but other genetic elements may also be considered for the explanation of the mechanism of resistance [4].

Recently, innovation of different and precise molecular techniques has played a big role in the detection of* mec*A gene, including DNA hybridization and polymerase chain reaction (PCR) [5].

Molecular amplification of the* mec*A gene is recognized as a benchmark to diagnose MRSA in the community as these genes are highly conserved among staphylococcal species [6]. The present project aimed to evaluate the usefulness of amplification of* mec*A gene and its reliability in the identification of MRSA strains.

## 2. Patients and Methods

### 2.1. Clinical Strains

Two hundred isolates of staphylococci were collected from subjects attending various hospitals and medical centers at Shendi City, Northern Sudan, after obtaining their informed consent. Clinical samples which included wound and ear swabs and urine and nasal secretions were collected from April 2013 to October 2014. Swabs samples were added in sterile tubes of Brain Heart Infusion Broth (HiMedia) while urine samples were inoculated on MacConkey's and blood agar and then all primary cultures were subcultured on Mannitol Salt Agar (ALPHA) and identified primarily by routine laboratory procedures which included microscopic morphology and biochemical tests including *β*-hemolysis on blood agar, catalase 3%, oxidase, urease, and DNase. Colonies grown were cultured into Nutrient Agar (ALPHA) for further testing [7, 8].

## 3. Antibiotic Susceptibility Testing

Susceptibility test was done for all the two hundred* S. aureus* isolates against the following antibiotics: oxacillin, penicillin, gentamicin, ampicillin, tetracycline, clindamycin, amoxicillin, linezolid, sulfamethoxazole-trimethoprim, and imipenem (HiMedia) by modified Kirby-Bauer technique based on NCCLS regulations [7, 8]. Furthermore,* E*-test was used to estimate the minimum inhibitory concentrations (MICs) for all MRSA isolates as instructed by the manufacturer.

### 3.1. Extraction of DNA

Bacterial DNA was isolated from all pure staphylococcal strains with the aid of ready kit from Thermo Scientific GeneJET Genomic, Lithuania.

### 3.2. Detection of* arc*C Gene

A single PCR assay targeting* S. aureus* species specific gene,* arc*C (Carbamate kinase gene, a determinant of* Staphylococcus aureus*), was adopted as described by Al-Abbas 2012 [6]; a 25 *µ*L reaction was prepared which contained 2.5 *µ*L 10x PCR buffer, 2.5 *µ*L MgCl_2_ (25 mM), 1 *µ*L dNTP mix (10 mM), 0.2 *µ*L of each primer, 0.5 *µ*L Taq polymerase (5 U/*µ*L), and 17.1 *µ*L distilled water. Then 1 *µ*L template DNA was added separately to each reaction tube with a final volume of 25 *µ*L/reaction.

The thermal profile was as follows: initial 5 minutes' denaturation step at 94°C for one cycle followed by repeating cycles of denaturation (30 seconds at 94°C), annealing (45 seconds at 55°C), and extension (40 seconds at 72°C) for 35 cycles, followed by a 5 minutes' final extension step at 72°C.

PCR product was visualized on 2% agarose gel and band size was compared to DNA marker (100 bp). Positive results of* Staphylococcus aureus* will produce a band of 356 bp for* arc*C gene.

### 3.3. Amplification of* mec*A Gene

*S. aureus* strains were subjected to PCR searching for the* mec*A gene according to Al-Abbas 2012 [6]. PCR protocol was adopted in 25 *µ*L volume which contains 1 U* Taq* polymerase and the buffer conditions recommended by the manufacturer (Promega). A PCR program was conducted with initial denaturation at 94°C for 5 min followed by 30 cycles of 94°C for 60 sec, 62°C for 30 sec, and 72°C for 35 sec ended with a final extension at 72°C for 10 min. Then, the PCR product was visualized under UV transilluminator on 2% agarose, and the following primers were used.

F: 5′-GTAGAAATGACTGAACGTCCGATGA-3′ and R: 5′-CCAATTCCACATTGTTTCGGTCTAA-3′. These produce a PCR amplicon of 310 base pairs.* S. aureus* reference strain EMRSA-15 was used as a positive control for the* mec*A while NCTC 6571 was used as negative control.

## 4. Results

### 4.1. Drug Susceptibility Testing

Of the total 200* S. aureus* isolated, 123 (61.5%) strains were defined as methicillin resistant (MRSA) and 77 (39.5%) were sensitive to methicillin (MSSA). Furthermore, all the study samples were tested against different antibiotics; the results are shown in Table 1.

### 4.2. Susceptibility to Methicillin (Oxacillin) (MICs)

**Figure 1:**
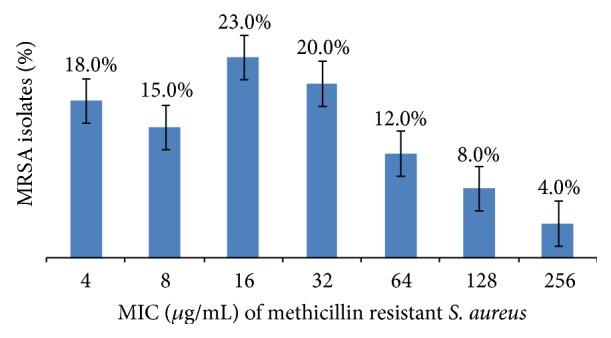
MRSA isolates with different MICs ranges.

The results obtained by* E*-test reflected that all the methicillin resistant* S. aureus* isolates were strongly resistant to methicillin with minimum inhibitory concentrations varying from 4 to 256 *μ*g/mL as illustrated in Figure 1.

### 4.3. MRSA Distribution among Different Clinical Samples

**Figure 2:**
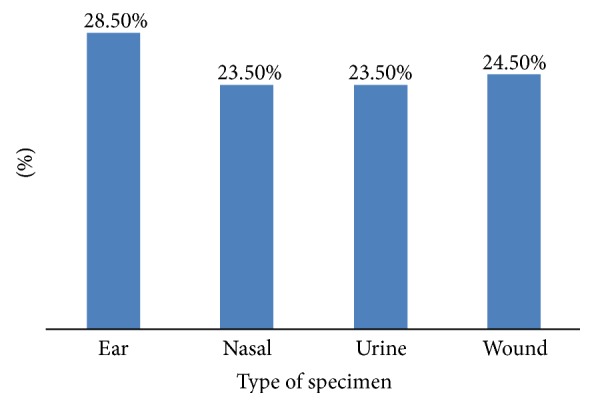
Frequency of MRSA isolates among different clinical samples.

Methicillin resistant* S. aureus* was isolated from different body sites as follows: 28.5, 24.5, 23.5, and 23.5% from ear, wound, urine, and nasal specimens, respectively (Figure 2).

### 4.4. Amplification of* arc*C Gene by PCR

**Figure 3:**
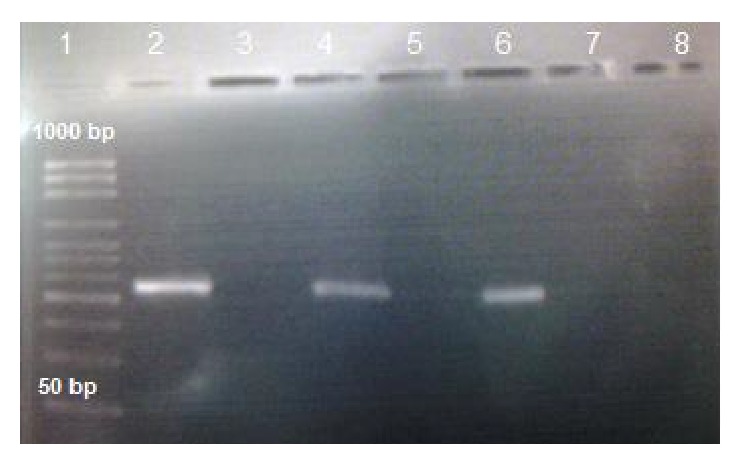
Amplicon of* arc*C gene; lane 1: molecular weight ladder; lane 2: positive control; lane 5: negative control; lanes 7 and 8: negative samples; lanes 4 and 6: positive samples, as indicated by the 356 bp PCR product.

All tested strains belonging to the staphylococcal species reflected positive results for the* arc*C gene with a band size of 356 bp as illustrated in Figure 3.

### 4.5. Amplification of* mec*A Gene

**Figure 4:**
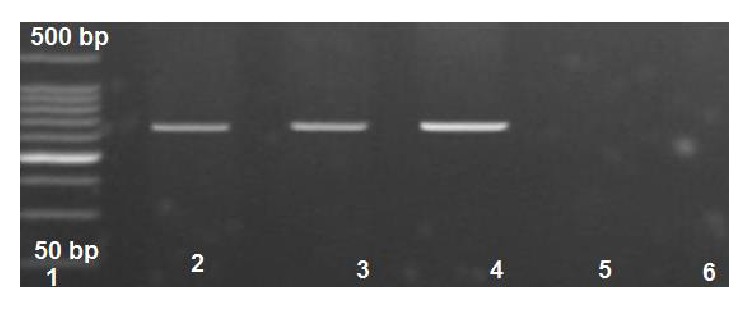
Amplicon of* mec*A gene; lane 1: 50 bp molecular weight ladder; lane 2: positive control; lanes 3 and 4 are tested isolates with positively amplified* mec*A as indicated by 310 bp PCR amplicon; lanes 5 and 6 are* mec*A negative (methicillin susceptible* S. aureus*).

The* mec*A gene was examined in all subjected methicillin resistant* S. aureus* (MRSA) isolates; 111/123 (90.2%) were* mec*A positive while the remaining 12/123 (9.8%) failed to produce the band of 310 bp specific for* mec*A gene. Moreover, all MSSA isolates (77) were* mec*A negative (Figure 4).

## 5. Discussion

Improving a consistent method for the early and precise diagnosis of MRSA as well as VRSA is still highly required. Such accomplishment will represent a vital part for controlling the spread of this pathogen in the community.

This study attempted to establish a map of the current situation of* S. aureus* and its resistance to the empirical drugs that are used to treat this pathogen in different hospitals and medical centers in Shandi City, Sudan. One of the major findings in this project is the high percentage of MRSA strains (61.5%), which had also revealed multiple resistance to various drugs. In addition to oxacillin, they are resistant to penicillin, ampicillin, gentamicin, and kanamycin. This high rate of resistance in clinical isolates was reported previously by many authors: 54% in Egypt [9], 57% in Jordan [10], 45.5% in Japan [11], 51% in Saudi Arabia [12], 61% in Taiwan [13], 61.8% in USA [14], and 69.4% in one report and 78.0% in another report from Sudan [15, 16].

Finding of* mec*A gene is the major evidence for the detection of MRSA isolate. This statement was approved by many researchers all over the world: in Sudan [15, 16], in Egypt [9], in Saudi Arabia [17], in Iran [18], in Iraq [10], in Japan [19], in Spain [20], in England [21, 22], in India [23], in Australia [24], and in USA [25]. However, our findings in this project suggested low burden of the* mec*A gene (90.2%); this may open the door to search for other intrinsic factors that may compete with* mec*A gene in producing resistance phenomenon in regions with high prevalence of MRSA. On the other hand, the absence of* mec*A gene within resistant staphylococcal isolates was listed worldwide [26–28]. Additionally, moderate methicillin resistance was observed in isolates that lacked the* mec*A gene mutations [29, 30]. Also a previous study in Nigeria reported the complete absence of five major SCCmec types and* mec*A genes as well as the gene product of PBP2a in isolates which were phenotypically MRSA suggesting a probability of hyperproduction of *β*-lactamase as a cause of the phenomenon [31]. Moreover, recently Ba and colleagues mentioned specific alterations in different amino acids present in protein binding proteins cascade (PBPs 1, 2, and 3) which may be the basis of resistance [32]. These alterations were found to include three amino acid substitutions which were identical and were present in PBPs 1, 2, and 3. Moreover, the same amino acid was found to have two other different substitutions in PBP1. Both the identical and different amino acid substitutions were observed in isolates from different multilocus types [32]. These findings provided clear evidence that there are mechanisms other than the presence of* mec*A gene responsible for beta-lactam resistance of MRSA and that molecular methods alone are not enough for confirmed characterization of MRSA isolates, a point that should be under consideration by regional and reference laboratories.

## 6. Conclusion

In conclusion, our findings indicate increasing prevalence of MRSA in Shendi area (61.5%), which represents an alarm for the health authorities putting into consideration the emergence of VRSA isolates in the community. In addition, PCR-based detection of MRSA is highly recommended. However, the absence of* mec*A gene in a considerable percentage of MRSA isolates requires investigating the alternative genetic possibilities related to the resistance phenomena.

## Figures and Tables

**Table 1:** Drug susceptibility pattern of the study isolates.

Antibiotic	MRSA (out of 123)	MSSA (out of 77)
	Number (%)	Number (%)
Methicillin	123 (100)	00 (0.0)
Penicillin	123 (100)	74 (96.0)
Ampicillin	123 (100)	74 (96.0)
Gentamicin	116 (94.0)	6 (8.0)
Kanamycin	113 (92.0)	23 (30.0)
Imipenem	69 (56.0)	20 (26.0)
Amoxicillin	64 (52.0)	23 (30.0)
Ciprofloxacin	106 (86.5)	17 (22.0)
Clindamycin	88 (71.3)	25 (32.0)
Linezolid	16 (13.0)	00 (0.0)
Sulfamethoxazole-trimethoprim	74 (60.0)	31 (40.0)
